# Pregnancy related risk perception in pregnant women, midwives & doctors: a cross-sectional survey

**DOI:** 10.1186/s12884-019-2467-4

**Published:** 2019-09-27

**Authors:** Suzanne Lee, Des Holden, Rebecca Webb, Susan Ayers

**Affiliations:** 1 School of Health Sciences, City University London, Northampton Square, London, EC1A 7QN UK; 2 Surrey and Sussex Healthcare NHS Trust, East Surrey Hospital, Canada Avenue, Redhill, Surrey, RH1 5RH UK

## Abstract

**Background:**

Risk perception in relation to pregnancy and birth is a complex process influenced by multiple personal, psychological and societal factors. Traditionally, the risk perception of healthcare professionals has been presented as more objective and authoritative than that of pregnant women. Doctors have been presented as more concerned with biomedical risk than midwives. Such dichotomies oversimplify and obscure the complexity of the process. This study examines pregnancy-related risk perception in women and healthcare professionals, and what women and professionals believe about each other’s risk perception.

**Methods:**

A cross sectional survey of set in UK maternity services. Participants were doctors working in obstetrics (*N* = 53), midwives (*N* = 59), pregnant women (*N* = 68). Participants were recruited in person from two hospitals. Doctors were also recruited online. Participants completed a questionnaire measuring the degree of perceived risk in various childbirth-related scenarios; and the extent to which they believed others agreed with them about the degree of risk generally involved in childbirth. Main outcome measures were the degree of risk perceived to the mother in baby in pregnancy scenarios, and beliefs about own perception of risk in comparison to their own group and other groups.

**Results:**

There were significant differences in total risk scores between pregnant women, doctors and midwives in perception of risk to the mother in 68/80 scenarios. Doctors most frequently rated risks lowest. Total scores for perceived risk to the baby were not significantly different. There was substantial variation within each group. There was more agreement on the ranking of scenarios according to risk. Each group believed doctors perceived most risk whereas actually doctors most frequently rated risks lowest. Each group incorrectly believed their peers rated risk similarly to themselves.

**Conclusions:**

Individuals cannot assume others share their perception of risk or that they make correct assessments regarding others’ risk perception. Further research should consider what factors are taken into account when making risk assessments,

**Electronic supplementary material:**

The online version of this article (10.1186/s12884-019-2467-4) contains supplementary material, which is available to authorized users.

## Background

Risk perception in relation to pregnancy and birth is a complex process based on multiple factors [1, 2]. It is influenced by factors pertaining to risk perception beyond the subject of childbirth and pregnancy-specific factors. Factors involved in general risk perception include the degree of perceived control involved in undertaking the risk activity, the ways in which information about the risk is presented, and the degree of trust placed in the source of the information [3–5]. Factors specific to pregnant women include the extent to which women view childbirth as benefitting from medical management, and common concerns for the wellbeing of their babies [6, 7].

The two main discourses within which childbirth can be understood also each have different approaches to risk. The biomedical model regards birth as inherently risky [8], whereas the social model regards pregnancy risk as a concept constructed from multiple cultural and personal factors [9]. Risk in the biomedical model is generally presented in terms of potential physiological outcomes whereas the social model recognises a more holistic definition encompassing potential threats to psychological and social wellbeing. In line with these discourses, views of maternity healthcare professionals have often been presented in a polarised fashion: typically doctors are presented as supporting the medical model and midwives the social model [10, 11]. Doctors’ education has frequently focussed on birth as a biomechanical process whereas midwives may consider it in more holistic terms [12, 13]. However, presenting these approaches as entrenched opposites oversimplifies the issue [14]. Such dichotomous depictions overlook the fact all healthcare professionals are likely to be aiming to provide high quality healthcare and ensure positive outcomes [15] and may well move along the spectrum between approaches [16].

Accounts of risk perception should also consider women’s perspectives. In contemporary Western culture, women’s understanding of birth has typically been portrayed as more subjective and less well-informed than that of professionals [17]. However, women’s perception of risk often transcends the medical and social models as they act from unique and individual perspectives of their own circumstances [18]. The portrayal of lay persons’ perceptions of risk in relation to birth has also been simplified and polarised, with professional perceptions of risk often characterised as objective and logical, and lay assessments as more emotion-based [19]. Again, such dichotomous terms obscure and oversimplify the differences and similarities of risk perception between lay and professional groups. Perception of risk by members of both groups will entail an assessment of numerical odds but also be contingent on personal experience, context and interactions with others [1].

However, research examining lay and medical risk perception does find differences. A review of quantitative risk perception research found little correlation between perceptions held by healthcare professionals and of pregnant women with regard to pregnancy and childbirth [20]. Qualitative research has similarly shown differences in the way professionals and women define concepts of risk and safety [21]. However, there is little research in this area and existing studies have used different criteria for assessing risk perception of professionals and women, making them difficult to compare and reinforcing the idea the groups assess risk in different ways.

The aim of this study is therefore to examine pregnancy-related risk perception in women and maternity healthcare professionals in contemporary English society using a unified assessment tool to enable direct comparison, and to examine what women and professionals believe about each other’s risk perception. This study will contribute to the understanding of risk perception and inform professionals seeking to understand awareness of risk and improve communication with pregnant women.

## Method

### Design

We conducted a cross-sectional study of risk perception in regard to pregnancy and childbirth by administering a questionnaire (see Additional file 1) to doctors, midwives and pregnant women.

### Participants

A sample of pregnant women, midwives and doctors was recruited from two NHS Trusts (organisations providing state-funded healthcare and utilised by the majority of the population) in South East England. Seventy-eight pregnant women were approached and 68 (87%) completed questionnaires. Fifty-nine midwives completed the questionnaire (16% of those approached). Initial uptake of the study was low among doctors (*n* = 18, 20% of those approached) so recruitment was extended for this group. The final sample for analyses was 68 pregnant women, 59 midwives and 53 doctors. Doctors and midwives were comparable in terms of time since qualification: doctors’ mean time 14.8 years (SD = 10.23; range 1 to 37 years); midwives mean time 12.71 years (SD = 9.9; range 1 month to 34 years; t (109) = 1.09, *p* = .28). The midwives worked in obstetric units and community settings; neither trust involved in the study has a midwifery-led unit.

Inclusion criteria for healthcare professionals were a recognised medical or midwifery qualification and currently working as a doctor or midwife in a UK maternity service. This allowed for participation by junior doctors not specialising in obstetrics but currently on an obstetric placement. Pregnant women had to be between 18 and 21 weeks pregnant and aged 18 or over to participate.

Recruitment occurred between June 2016 and April 2017. Participants were initially recruited through two NHS trusts in South East England. However, difficulties recruiting doctors to the study meant it was necessary to extend recruitment to include any doctor practising obstetrics in the UK. These additional participants were recruited via social media and completed the study online.

Pregnant women were approached by a researcher while waiting for their mid-pregnancy anomaly scan. All women in the UK are offered this scan so this time was chosen to reach the maximum number of women. They were given verbal and written information about the study and reassurance about confidentiality. If they agreed to participate, they completed the questionnaire straight away and returned it to the researcher. Information about the study was placed in the maternity departments for doctors and midwives along with copies of the questionnaire for them to complete at their convenience. Researchers also visited the maternity units to provide further information and approach staff in person. Consent, agreed as part of the ethics approval for the study, was considered indicated by completion of the questionnaire. All participants received information about the study explaining the procedure.

### Measures

The questionnaire consisted of two elements. The first measured risk in relation to pregnancy and childbirth scenarios (80 items) and was adapted from the work of Gray [22], a who used a similar scale to measure risk perception in relation to hospitalisation in pregnancy. Each item in the first element briefly described a pregnancy or birth scenario, e.g. ‘a woman who has a minor postpartum haemorrhage at home’, ‘a woman giving birth in a birthpool in hospital’. Participants were asked to rate how much risk they perceived each scenario represented to the wellbeing of (i) the mother and (ii) the fetus/baby. Participants were provided with a definition of risk as ‘a possible degree of threat to physical or psychological wellbeing’. This definition was intended to allow participants to formulate their own interpretations of scenarios as far as possible. Three obstetricians, three midwives and six pregnant women assisted in the development and piloting of the questionnaire to ensure user acceptability and face validity.

All participants received the same questionnaire but the version for pregnant women also included definitions of medical terms (e.g. shoulder dystocia, pre-eclampsia, etc.) taken from the NHS Choices or Royal College of Obstetricians and Gynaecologists websites. Participants indicated the degree of risk perceived using a visual analogue scale: a 100 mm line representing a continuum from ‘minimal risk’ to ‘extreme risk’. The distance from the beginning of the scale to participants’ marks was measured and the resulting millimetre measurement became the risk score for each question (minimum score = 0; maximum score = 100). Risks to mother and baby were scored separately, providing total possible risk scores of 8000 for risk to mother and 8000 for risk to baby (80 scenarios, maximum score 100 per scenario).

The second element looked at participants’ comparison of their own perceived risk with other groups (3 items). Participants were asked to compare the extent to which they believed others agreed with them about the degree of risk generally involved in pregnancy and birth. A seven point Likert scale was used ranging from (1) ‘they think it is a lot less risky than I do’ to (7) ‘they think it is a lot more risky than I do’. A score of 4 therefore indicated they believed the comparison group agreed with them about the degree of risk. Participants first compared themselves with members of their own group (other doctors, midwives or pregnant women) and then with the remaining two groups.

The primary outcomes were degree of perceived risk, and perception of own sense of risk in comparison to others in their own group and other groups.

Sample size calculations based on the likelihood of detecting a medium effect size estimated a sample of 53 doctors, 53 midwives and 53 pregnant women was needed for the study to be adequately powered to find a difference in the primary outcomes (power = 0.8, significance = .05).

Doctors and midwives were also asked how long they had held their medical or midwifery qualification.

### Analysis

Analysis was conducted using SPSS version 23 [23]. Kolmogorov-Smirnov and Levene’s tests were conducted to test for normal distribution and homogeneity of variance. ANOVA was used to test for differences in total scores between groups. The data were found to be skewed for scores on individual items so the non-parametric Kruskal-Wallis test was used to test for differences between the groups.

## Results

Doctors’, midwives’ and pregnant women’s total mean scores of overall perceived risk to the mother and baby are shown in Table 1.

**Table 1:** Mean scores of overall perceived risk

	Possible range	Doctors Mean (SD)	Midwives Mean (SD)	Pregnant women Mean (SD)	*p* value
Perceived risk to the mother	0–8000	3127.64 (1338.95)	3776.94 (708.03)	3741.76 (849.09)	.018
Perceived risk to baby	0–8000	3305.77 (1272.45)	3648.88 (717.27)	3615.15 (816.59)	.215

Overall mean risk scores were low to moderate for mother and baby, ranging from 3127.64–3776.94 from a possible score of 8000. Doctors reported the lowest mean scores. There were significant differences between groups for risk to the mother but not for risk to the baby. Post hoc tests revealed significant differences for perceived risk to the mother between doctors and midwives (mean difference 559.3, SE 211.05, *p* = .02) and doctors and pregnant women (mean difference 524.13, SE 208.01, *p* = .03) but not between midwives and pregnant women (mean difference 35.18. SE 183.64, *p* = .98).

Across individual risk scenarios however, there was little agreement between the groups on the perceived degree of risk (see Additional file 2). Regarding risk to the mother, 68 scenarios (85%) showed significant differences between the groups (*p* < .05) and only 12 (15%) showed no significant differences in risk scores (*p* = .077–.576). There was also little agreement in scores on risk to the baby: 58 scenarios (73%) showed a significant difference between the groups (*p* < .05) and 22 scenarios (27%) showed no significant differences (*p* = .051–.724). Of the 12 scenarios which showed no difference in risk scores for risk to the mother, 11 of these also showed no difference for risk to the baby.

In scenarios where there was a difference between risk scores, doctors consistently rated the risk lowest. With regards to risks to the mother, doctors rated the risks lowest for the majority of scenarios where there was a significant difference between groups (56 times out of 68). There was a similar picture with scores of risk to the baby where doctors rated the risks lowest for the majority of scenarios where there was a significant difference between groups (41 times out of 58). Pregnant women rated the most scenarios the highest (mothers’ risk 39 of 68; babies’ risk 30 of 58). Midwives rated slightly fewer scenarios highest (mothers’ risk 32 of 68; babies’ risk 28 of 58). Doctors’ ratings of risk were highest only once for mothers’ risk and once for babies’ risk.

However, although there was variation between groups in the risk scores assigned to the mothers and babies in the scenarios, there were similarities across the groups when the scenarios were ranked in order of degree of perceived risk. The 10 highest ranking scenarios are shown in Table 2 for risk to mothers and Table 3 for risk to babies. Six scenarios were consistently rated highest for risk to the mother by participants in all three groups. Four scenarios were consistently rated highest risk to the baby by participants in all three groups. Thus while there was frequent disagreement about the degree of risk posed in each scenario, there was more consensus about the scenarios that posed the greatest risk to mothers and babies.

**Table 2:** Highest ranking scenarios for risk to mother

Scenario	Ranking		
	Doctors	Midwives	Pregnant women
A pregnant woman with severe pre-eclampsia not receiving any antenatal care	1	2	2
A woman who has a major postpartum haemorrhage at home	2	1	1
A woman bleeding heavily at 37 weeks of pregnancy	3	7	7
A pregnant woman experiencing domestic violence	4	2	5
A woman who chooses to give birth at home alone	5	4	4
A pregnant woman with mild pre-eclampsia not receiving any antenatal care	7	7	7

**Table 3:** Highest ranking scenarios for risk to baby

Scenario	Ranking		
	Doctors	Midwives	Pregnant women
A pregnant woman with severe pre-eclampsia not receiving any antenatal care.	2	2	4
A woman with a shoulder dystocia at home.	3	4	7
A woman who gives birth at 26 weeks of pregnancy.	4	3	2
A woman bleeding heavily at 24 weeks of pregnancy.	6	5	1

Mean scores for participants’ estimations of how their peers and members of the other groups would rate risk in comparison to their own ratings are displayed in Table 4. Each group believed their peers agreed with them about the degree of risk generally involved in pregnancy and childbirth. Doctors believed midwives perceived birth as a little less risky than they did and pregnant women perceived it as somewhat less risky. Midwives believed doctors perceived pregnancy and birth as somewhat more risky and pregnant women as a little less risky than they did. Pregnant women believed doctors agreed with them about the degree of risk involved in pregnancy and birth. Their mean score for comparison with midwives fell between midwives believing birth is a little less risky and agreeing with the degree of risk.

**Table 4:** Comparisons of risk perception

		Compared self with		
		Doctors	Midwives	Pregnant women
Participants	Doctors	4 (Agree with me)	3 (A little less risky than I do)	2 (Somewhat less risky than I do)
	Midwives	6 (Somewhat more risky than I do)	4 (Agree with me)	3 (A little less risky than I do)
	Pregnant women	4 (Agree with me)	3.5 (A little less risky than I do/agree with me)	4 (Agree with me)

Key: Think pregnancy and birth are: 1 = A lot less risky than I do; 2 = Somewhat less risky than I do; 3 = A little less risky than I do; 4 = Agree with me about the degree of risk involved; 5 = A little more risky than I do; 6 = Somewhat more risky than I do; 7 = A lot more risky than I do.

## Discussion

This study aimed to examine risk perception in relation to pregnancy and childbirth in pregnant women and maternity healthcare professionals in contemporary English society, and to examine what pregnant women and professionals believe about the risk perceptions of each other. Results showed there were differences in risk perception in relation to pregnancy and birth within and between pregnant women, doctors and midwives. When rating risk of different scenarios to mothers, there were significant differences between pregnant women, doctors and midwives for 68 out of 80 pregnancy-related scenarios. This was less marked when rating risks for babies where overall mean risk scores were not significantly different between groups, but there were differences in the scores of 58 out of 80 scenarios. While assessments of the degree of risk pertaining to scenarios differed, there was more agreement on the ranking of scenarios according to risk. Interestingly, when comparing their own ratings of risk to others, each group incorrectly believed their peers rated risk similarly to themselves. These beliefs were not supported by the first part of the study. The mean risk score for each group, and in particular the doctors, had large standard deviations, indicating a wide range of scores within groups (see Table 1). Each group also believed doctors perceived most risk in pregnancy and birth. However, where there were significant differences in scores for individual scenarios, where doctors consistently rated the risks lower than women and midwives.in The results of this study are consistent with previous research showing pregnant women think about risk differently to healthcare professionals. It showed pregnant women had the highest risk perception scores. Most pregnant women experience some fears regarding risk, especially concerning the birth process and the wellbeing of their babies [24]. While this may appear concerning, these fears are not necessarily perceived negatively by women however and, though common, are often not perceived as very intense or intrusive. They may be viewed as an integral part of pregnancy which confers an element of protection by motivating women to speak out about their concerns [25]. Thus while women acknowledge pregnancy and birth may involve a degree of risk they also utilise psychological strategies to manage these concerns and may tolerate greater degrees of risk than professionals recommend, or would be prepared to tolerate for themselves, if they believe doing so will result in a better outcome for themselves and their babies [26, 27]. Within the professionals’ risk assessments, midwives also rated risks to mothers significantly higher than doctors. While midwifery care is based on the assumption and promotion of normality in pregnancy, much midwifery activity is focussed on concerns regarding the abnormal so creating a degree of tension and disjunction between rhetoric and practice [28, 29].

This study found differences between the risk perception of pregnant women and healthcare professionals. It was beyond the scope of the study to investigate potential reasons for these differences but they have traditionally been attributed to women’s more subjective, less knowledge-based understanding of risk [17, 19]. However, such a distinction may be misplaced; emotion, imagination and intuition are components of all perceptions of risk and so are likely to influence women and professionals alike alongside an assessment of objective knowledge [30, 31]. Healthcare professionals have been shown to consider the degree of the patient’s anxiety, their own clinical experience, particularly that based on borderline or especially interesting cases and negative outcomes, and the opinions of trusted colleagues when making decisions regarding risk [32, 33]. They actively interpret evidence-based guidelines according to their own and the patient’s individual circumstances rather than follow them absolutely. Rather than differentiating between lay (subjective, inferior) and professional (objective, authoritative) understanding of risk, it may be more appropriate to acknowledge both groups rely on different types of understanding: systematic, intuitive, experiential and tacit, when making judgements about risk [34]. Further research is needed to establish how maternity professionals combine subjective and objective elements of risk perception. It may also be the case that doctors become habituated to situations involving greater degrees of risk as they make up the majority of their practice and utilise their obstetric skills.

The midwives and doctors in this study demonstrated some differences in risk perception. Perceived differences in approach to risk have been shown to be a factor which can lead to tension between maternity care professionals and feelings of lack of trust and support [35]. Such tension maybe interpreted as conflict by service users and cause them some anxiety [36]. The members of each group in this study believed their peers agreed with them about the degree of risk involved in pregnancy and childbirth, although the results did not support this belief. In all communication, individuals typically overestimate the extent to which others share their beliefs and interpretation of events [37]. This false consensus effect is particularly evident in situations deemed important, in this instance healthcare. These factors can lead to misinterpreted communication and conflict [38] so maternity care professionals should be aware of their potential influence in communication with members of their own discipline as well as others and pregnant women.

This study demonstrates risk perception in relation to pregnancy and birth is a complex and individual process. It challenges the stereotypical depictions of doctors and midwives in regard to their perception of risk. This has implications for how professionals perceive their own and others’ roles, and how they communicate with each other and with women in their care. Traditional distinctions between the quality of professional and lay approaches to risk oversimplify the process and may devalue women’s understanding of their circumstances and contribution to their car [14]. Recognising all parties have a common goal and speaking openly about factors influencing risk perception may enhance relationships with women and colleagues [39]. This in turn has been demonstrated to have a positive effect on outcomes for women and babies [40].

This is the first study to explore the risk perception of doctors, midwives and pregnant women using a comparable measure. It adds to the understanding of how the different groups assess risk, their beliefs about the risk perception of their peers and others, and has implications regarding communication within and between groups. It will contribute to the understanding of risk perception and the conceptualisation of risk. It will increase knowledge of risk perception in different groups and their beliefs about each other, and challenge unhelpful stereotypes. It will inform professionals working in maternity care and seeking to understand awareness of risk and improve communication with pregnant women, and provide information for women utilising maternity care.

Strengths of this study include using the same measures for each group to allow direct comparisons of risk perception across groups. The study was developed with input from maternity service users, obstetricians and midwives, ensuring its credibility and acceptability. The sampling strategy ensured the inclusion of pregnant women with different degrees and experiences of risk in their own pregnancies and healthcare professionals working in different areas of maternity care with varying lengths of time qualified to provide a range of potential perspectives on risk. A limitation of the study was the difficulty recruiting doctors and need to include participants recruited online. The final sample therefore included doctors from other areas so the group was potentially more heterogeneous and self-selected than were the groups of pregnant women and midwives. The relatively small numbers of doctors and midwives who ultimately participated in the study mean further work is needed to establish how representative their perceptions of risk are and the extent to which the findings are generalisable. Although efforts were made to ensure face validity, the questionnaire has not been formally validated so further work is needed to establish the extent to which it accurately represents perception of risk and the extent to which these perceptions manifest in behaviour. Finally, between-group differences were tested for all 80 individual scenarios and multiple comparisons increase the probability that some of the significant findings occurred by chance. It is therefore important to replicate and extend this research.

## Conclusions

Assessment of risk is a complex activity comprised of objective and subjective elements. All individuals making an assessment of risk will rely on these elements to differing extents depending on the particular context. Individuals cannot assume others share their perception of risk or that they make correct assessments regarding the risk perception of others.

Healthcare professionals should be aware of these considerations when communicating with pregnant women, and also with other professionals. Communication with women should include sensitive and respectful exploration of their understanding and perception of risk and safety, and acknowledgement of any anxieties. When communicating with members of their own discipline, professionals should not make assumptions that colleagues will share their perceptions. They should be mindful of the subjectivity of their own risk assessments and factors which may influence their perceptions rather than attempting to eliminate or deny the effects of some of these influences. They should challenge their own and others’ stereotypical assumptions about the perception of risk in other disciplines, and ensure interdisciplinary communication is based on professional respect and genuine understanding.

Further research is needed to consider what factors professionals and pregnant women take into account when making risk assessments in relation to pregnancy and birth and how they use these factors to formulate their assessments. It should also examine which elements of pregnancy and birth are perceived as most risky, what it is about them which causes concern, and the extent to which evidence exists to support these perceptions. Qualitative research should explore healthcare professionals’ and pregnant women’s understanding and awareness of their perceptions of risk. Research is needed to establish how perception of risk influences professionals’ communication with women, the barriers to effective risk communication, and how this communication can be improved. Research into interprofessional working should explore how stereotypical expectations about the risk perception of other groups arise, can be challenged, and communication between disciplines improved.

## Additional files

Additional file 1: Risk Questionnaire (doctors’ version). (DOCX 34 kb)

Additional file 2: Differences between perceived risk ratings by pregnant women, midwives and doctors for individual scenarios. (DOCX 14 kb)

## Data Availability

The datasets used and/or analysed during the current study are available from the corresponding author on reasonable request.
