# Supplementary material for: Pregnancy related risk perception in pregnant women, midwives & doctors: a cross-sectional survey
Source: BMC Pregnancy Childbirth. 2019 Sep 27;19:335. doi: 10.1186/s12884-019-2467-4 (PMC6764151; doi:10.1186/s12884-019-2467-4)
Supplement: Supplementary file 1 — Risk Questionnaire (doctors’ version). (DOCX 34 kb) [file 12884_2019_2467_MOESM1_ESM.docx]

**Supplementary File 1: Risk Questionnaire (doctors’ version)**

Please state your job title…………………………………………….………

How long have you been qualified as a doctor?..............................................

Please answer the following questions by marking an X along the line.

1. How anxious do you feel at the moment? The line represents a scale from feeling no anxiety to feeling extreme anxiety.

…………………………………………………………….. Not at all Extremely anxious anxious

1. How risky do you think the following activities are? Please mark an X along the line to show the degree of risk for each activity. The left hand end of the line represents virtually no risk and the right hand end of the line represents a very high risk.
2. Crossing a busy motorway whilst blindfolded.

……………………………………………………………..

Minimal Extreme risk risk

1. Sitting in an armchair.

……………………………………………………………..

Minimal Extreme risk risk

The following statements are brief scenarios which describe various aspects of pregnancy and childbirth. Please indicate the degree of risk you believe each one poses to the woman and baby involved. Risk means a possible degree of threat to physical or psychological wellbeing.

Please mark an X along the line to show the degree of risk in each scenario. The left hand end of the line represents virtually no risk and the right hand end of the line represents a very high risk.

1. A woman in labour being cared for by an experienced midwife.

Risk to the woman

……………………………………………………………..

Minimal Extreme risk risk

Risk to the baby

……………………………………………………………..

Minimal Extreme risk risk

1. A woman who gives birth on all fours.

Risk to the woman

……………………………………………………………..

Minimal Extreme risk risk

Risk to the baby

……………………………………………………………..

Minimal Extreme risk risk

1. A woman having a caesarean section because her labour did not progress.

Risk to the woman

……………………………………………………………..

Minimal Extreme risk risk

Risk to the baby

……………………………………………………………..

Minimal Extreme risk risk

1. A pregnant woman with a supportive partner.

Risk to the woman

……………………………………………………………..

Minimal Extreme risk risk

Risk to the baby

……………………………………………………………..

Minimal Extreme risk risk

1. A pregnant woman experiencing domestic violence.

Risk to the woman

……………………………………………………………..

Minimal Extreme risk risk

Risk to the baby

……………………………………………………………..

Minimal Extreme risk risk

1. A woman who chooses to give birth with just her partner present at home.

Risk to the woman

……………………………………………………………..

Minimal Extreme risk risk

Risk to the baby

……………………………………………………………..

Minimal Extreme risk risk

1. A woman who has a minor postpartum haemorrhage at home.

Risk to the woman

……………………………………………………………..

Minimal Extreme risk risk

Risk to the baby

……………………………………………………………..

Minimal Extreme risk risk

1. A pregnant 13 year old.

Risk to the woman

……………………………………………………………..

Minimal Extreme risk risk

Risk to the baby

……………………………………………………………..

Minimal Extreme risk risk

1. A pregnant woman who does not attend any antenatal care.

Risk to the woman

……………………………………………………………..

Minimal Extreme risk risk

Risk to the baby

……………………………………………………………..

Minimal Extreme risk risk

1. A woman with a complicated labour being cared for a by a junior doctor.

Risk to the woman

……………………………………………………………..

Minimal Extreme risk risk

Risk to the baby

……………………………………………………………..

Minimal Extreme risk risk

1. A pregnant woman who is financially wealthy.

Risk to the woman

……………………………………………………………..

Minimal Extreme risk risk

Risk to the baby

……………………………………………………………..

Minimal Extreme risk risk

1. A woman with a shoulder dystocia in hospital.

Risk to the woman

……………………………………………………………..

Minimal Extreme risk risk

Risk to the baby

……………………………………………………………..

Minimal Extreme risk risk

1. A woman bleeding heavily at 37 weeks of pregnancy.

Risk to the woman

……………………………………………………………..

Minimal Extreme risk risk

Risk to the baby

……………………………………………………………..

Minimal Extreme risk risk

1. A pregnant woman with mild pre-eclampsia not receiving any antenatal care.

Risk to the woman

……………………………………………………………..

Minimal Extreme risk risk

Risk to the baby

……………………………………………………………..

Minimal Extreme risk risk

1. A woman giving birth in a birthpool in hospital.

Risk to the woman

……………………………………………………………..

Minimal Extreme risk risk

Risk to the baby

……………………………………………………………..

Minimal Extreme risk risk

1. A woman with a complicated pregnancy being cared for by a junior doctor.

Risk to the woman

……………………………………………………………..

Minimal Extreme risk risk

Risk to the baby

……………………………………………………………..

Minimal Extreme risk risk

1. A woman in labour being cared for by a newly qualified midwife.

Risk to the woman

……………………………………………………………..

Minimal Extreme risk risk

Risk to the baby

……………………………………………………………..

Minimal Extreme risk risk

1. A woman who is very anxious during her pregnancy.

Risk to the woman

……………………………………………………………..

Minimal Extreme risk risk

Risk to the baby

……………………………………………………………..

Minimal Extreme risk risk

1. A woman bleeding heavily at 24 weeks of pregnancy.

Risk to the woman

……………………………………………………………..

Minimal Extreme risk risk

Risk to the baby

……………………………………………………………..

Minimal Extreme risk risk

1. A pregnant 38 year old.

Risk to the woman

……………………………………………………………..

Minimal Extreme risk risk

Risk to the baby

……………………………………………………………..

Minimal Extreme risk risk

1. A woman who has a minor postpartum haemorrhage in hospital.

Risk to the woman

……………………………………………………………..

Minimal Extreme risk risk

Risk to the baby

……………………………………………………………..

Minimal Extreme risk risk

1. A woman with a shoulder dystocia at home.

Risk to the woman

……………………………………………………………..

Minimal Extreme risk risk

Risk to the baby

……………………………………………………………..

Minimal Extreme risk risk

1. A woman who begins her pregnancy 3 stone (19kg) overweight.

Risk to the woman

……………………………………………………………..

Minimal Extreme risk risk

Risk to the baby

……………………………………………………………..

Minimal Extreme risk risk

1. A woman in her first pregnancy giving birth in hospital. The pregnancy has been straightforward.

Risk to the woman

……………………………………………………………..

Minimal Extreme risk risk

Risk to the baby

……………………………………………………………..

Minimal Extreme risk risk

1. A woman with a cord prolapse at home.

Risk to the woman

……………………………………………………………..

Minimal Extreme risk risk

Risk to the baby

……………………………………………………………..

Minimal Extreme risk risk

1. A woman who plans to give birth vaginally to a breech baby at home.

Risk to the woman

……………………………………………………………..

Minimal Extreme risk risk

Risk to the baby

……………………………………………………………..

Minimal Extreme risk risk

1. A woman whose labour is induced.

Risk to the woman

……………………………………………………………..

Minimal Extreme risk risk

Risk to the baby

……………………………………………………………..

Minimal Extreme risk risk

1. A woman who has an epidural in labour.

Risk to the woman

……………………………………………………………..

Minimal Extreme risk risk

Risk to the baby

……………………………………………………………..

Minimal Extreme risk risk

1. A woman in her second pregnancy giving birth in hospital. The pregnancy has been straightforward.

Risk to the woman

……………………………………………………………..

Minimal Extreme risk risk

Risk to the baby

……………………………………………………………..

Minimal Extreme risk risk

1. A woman who gives birth lying on her back.

Risk to the woman

……………………………………………………………..

Minimal Extreme risk risk

Risk to the baby

……………………………………………………………..

Minimal Extreme risk risk

1. A woman who gives birth at 26 weeks of pregnancy.

Risk to the woman

……………………………………………………………..

Minimal Extreme risk risk

Risk to the baby

……………………………………………………………..

Minimal Extreme risk risk

1. A woman with a complicated labour being cared for by a consultant obstetrician.

Risk to the woman

……………………………………………………………..

Minimal Extreme risk risk

Risk to the baby

……………………………………………………………..

Minimal Extreme risk risk

1. A woman in her first pregnancy giving birth at home. The pregnancy has been straightforward.

Risk to the woman

……………………………………………………………..

Minimal Extreme risk risk

Risk to the baby

……………………………………………………………..

Minimal Extreme risk risk

1. A woman who breastfeeds her baby.

Risk to the woman

……………………………………………………………..

Minimal Extreme risk risk

Risk to the baby

……………………………………………………………..

Minimal Extreme risk risk

1. A woman who has a major postpartum haemorrhage at home.

Risk to the woman

……………………………………………………………..

Minimal Extreme risk risk

Risk to the baby

……………………………………………………………..

Minimal Extreme risk risk

1. A woman who gives birth standing up.

Risk to the woman

……………………………………………………………..

Minimal Extreme risk risk

Risk to the baby

……………………………………………………………..

Minimal Extreme risk risk

1. A woman with a cord prolapse in hospital.

Risk to the woman

……………………………………………………………..

Minimal Extreme risk risk

Risk to the baby

……………………………………………………………..

Minimal Extreme risk risk

1. A pregnant woman who is financially poor.

Risk to the woman

……………………………………………………………..

Minimal Extreme risk risk

Risk to the baby

……………………………………………………………..

Minimal Extreme risk risk

1. A woman who develops diabetes in pregnancy and follows the advice of the hospital healthcare team regarding diet and insulin.

Risk to the woman

……………………………………………………………..

Minimal Extreme risk risk

Risk to the baby

……………………………………………………………..

Minimal Extreme risk risk

1. A woman who gives birth at 34 weeks of pregnancy.

Risk to the woman

……………………………………………………………..

Minimal Extreme risk risk

Risk to the baby

……………………………………………………………..

Minimal Extreme risk risk

1. A woman having a caesarean section because she wants to plan her baby’s birth date.

Risk to the woman

……………………………………………………………..

Minimal Extreme risk risk

Risk to the baby

……………………………………………………………..

Minimal Extreme risk risk

1. A pregnant woman receiving antenatal care from an experienced community midwife.

Risk to the woman

……………………………………………………………..

Minimal Extreme risk risk

Risk to the baby

……………………………………………………………..

Minimal Extreme risk risk

1. A woman who plans to give birth vaginally to a breech baby in hospital.

Risk to the woman

……………………………………………………………..

Minimal Extreme risk risk

Risk to the baby

……………………………………………………………..

Minimal Extreme risk risk

1. A pregnant woman who attends regular antenatal appointments.

Risk to the woman

……………………………………………………………..

Minimal Extreme risk risk

Risk to the baby

……………………………………………………………..

Minimal Extreme risk risk

1. A pregnant woman with severe pre-eclampsia not receiving any antenatal care.

Risk to the woman

……………………………………………………………..

Minimal Extreme risk risk

Risk to the baby

……………………………………………………………..

Minimal Extreme risk risk

1. A pregnant 45 year old.

Risk to the woman

……………………………………………………………..

Minimal Extreme risk risk

Risk to the baby

……………………………………………………………..

Minimal Extreme risk risk

1. A woman who is depressed during her pregnancy.

Risk to the woman

……………………………………………………………..

Minimal Extreme risk risk

Risk to the baby

……………………………………………………………..

Minimal Extreme risk risk

1. A woman who bottle-feeds her baby.

Risk to the woman

……………………………………………………………..

Minimal Extreme risk risk

Risk to the baby

……………………………………………………………..

Minimal Extreme risk risk

1. A woman having a caesarean section because her obstetrician has recommended it.

Risk to the woman

……………………………………………………………..

Minimal Extreme risk risk

Risk to the baby

……………………………………………………………..

Minimal Extreme risk risk

1. A woman planning a vaginal birth of twins at home.

Risk to the woman

……………………………………………………………..

Minimal Extreme risk risk

Risk to the baby

……………………………………………………………..

Minimal Extreme risk risk

1. A woman who begins her pregnancy 1stone (6.4kg) overweight.

Risk to the woman

……………………………………………………………..

Minimal Extreme risk risk

Risk to the baby

……………………………………………………………..

Minimal Extreme risk risk

1. A woman who has a caesarean section because her baby is breech.

Risk to the woman

……………………………………………………………..

Minimal Extreme risk risk

Risk to the baby

……………………………………………………………..

Minimal Extreme risk risk

1. A pregnant woman with severe pre-eclampsia being treated in hospital.

Risk to the woman

……………………………………………………………..

Minimal Extreme risk risk

Risk to the baby

……………………………………………………………..

Minimal Extreme risk risk

1. A woman who has a forceps delivery.

Risk to the woman

……………………………………………………………..

Minimal Extreme risk risk

Risk to the baby

……………………………………………………………..

Minimal Extreme risk risk

1. A pregnant asylum seeker.

Risk to the woman

……………………………………………………………..

Minimal Extreme risk risk

Risk to the baby

……………………………………………………………..

Minimal Extreme risk risk

1. A pregnant woman who does not have many friends.

Risk to the woman

……………………………………………………………..

Minimal Extreme risk risk

Risk to the baby

……………………………………………………………..

Minimal Extreme risk risk

1. A woman in labour who does not speak English.

Risk to the woman

……………………………………………………………..

Minimal Extreme risk risk

Risk to the baby

……………………………………………………………..

Minimal Extreme risk risk

1. A woman with a complicated pregnancy being cared for by a consultant obstetrician.

Risk to the woman

……………………………………………………………..

Minimal Extreme risk risk

Risk to the baby

……………………………………………………………..

Minimal Extreme risk risk

1. A woman giving birth in a birthpool at home.

Risk to the woman

……………………………………………………………..

Minimal Extreme risk risk

Risk to the baby

……………………………………………………………..

Minimal Extreme risk risk

1. A woman with a retained placenta at home following a homebirth.

Risk to the woman

……………………………………………………………..

Minimal Extreme risk risk

Risk to the baby

……………………………………………………………..

Minimal Extreme risk risk

1. A woman who experiences postnatal depression after giving birth.

Risk to the woman

……………………………………………………………..

Minimal Extreme risk risk

Risk to the baby

……………………………………………………………..

Minimal Extreme risk risk

1. A pregnant woman without a partner.

Risk to the woman

……………………………………………………………..

Minimal Extreme risk risk

Risk to the baby

……………………………………………………………..

Minimal Extreme risk risk

1. A woman planning a vaginal birth of twins in hospital.

Risk to the woman

……………………………………………………………..

Minimal Extreme risk risk

Risk to the baby

……………………………………………………………..

Minimal Extreme risk risk

1. A woman who experiences the ‘baby blues’ after giving birth.

Risk to the woman

……………………………………………………………..

Minimal Extreme risk risk

Risk to the baby

……………………………………………………………..

Minimal Extreme risk risk

1. A woman who has a Syntocinon drip in labour.

Risk to the woman

……………………………………………………………..

Minimal Extreme risk risk

Risk to the baby

……………………………………………………………..

Minimal Extreme risk risk

1. A woman who chooses to give birth alone at home.

Risk to the woman

……………………………………………………………..

Minimal Extreme risk risk

Risk to the baby

……………………………………………………………..

Minimal Extreme risk risk

1. A pregnant woman who has close supportive friends.

Risk to the woman

……………………………………………………………..

Minimal Extreme risk risk

Risk to the baby

……………………………………………………………..

Minimal Extreme risk risk

1. A pregnant woman with mild pre-eclampsia having regular appointments with her midwife and obstetrician.

Risk to the woman

……………………………………………………………..

Minimal Extreme risk risk

Risk to the baby

……………………………………………………………..

Minimal Extreme risk risk

1. A woman planning a homebirth who gives birth alone because her labour is so fast and the midwife has not arrived.

Risk to the woman

……………………………………………………………..

Minimal Extreme risk risk

Risk to the baby

……………………………………………………………..

Minimal Extreme risk risk

1. A woman who has a major postpartum haemorrhage in hospital.

Risk to the woman

……………………………………………………………..

Minimal Extreme risk risk

Risk to the baby

……………………………………………………………..

Minimal Extreme risk risk

1. A woman who develops diabetes in pregnancy and does not follow the advice of the hospital healthcare team regarding her diet.

Risk to the woman

……………………………………………………………..

Minimal Extreme risk risk

Risk to the baby

……………………………………………………………..

Minimal Extreme risk risk

1. A woman who experiences post-traumatic stress disorder after giving birth.

Risk to the woman

……………………………………………………………..

Minimal Extreme risk risk

Risk to the baby

……………………………………………………………..

Minimal Extreme risk risk

1. A woman having a caesarean section for twins.

Risk to the woman

……………………………………………………………..

Minimal Extreme risk risk

Risk to the baby

……………………………………………………………..

Minimal Extreme risk risk

1. A pregnant 17 year old.

Risk to the woman

……………………………………………………………..

Minimal Extreme risk risk

Risk to the baby

……………………………………………………………..

Minimal Extreme risk risk

1. A woman who develops diabetes in pregnancy and does not follow the advice of the hospital healthcare team regarding taking insulin.

Risk to the woman

……………………………………………………………..

Minimal Extreme risk risk

Risk to the baby

……………………………………………………………..

Minimal Extreme risk risk

1. A woman with a retained placenta following a hospital birth.

Risk to the woman

……………………………………………………………..

Minimal Extreme risk risk

Risk to the baby

……………………………………………………………..

Minimal Extreme risk risk

1. A woman in her second pregnancy giving birth at home. The pregnancy has been straightforward.

Risk to the woman

……………………………………………………………..

Minimal Extreme risk risk

Risk to the baby

……………………………………………………………..

Minimal Extreme risk risk

1. A woman who has a ventouse delivery.

Risk to the woman

……………………………………………………………..

Minimal Extreme risk risk

Risk to the baby

……………………………………………………………..

Minimal Extreme risk risk

1. A woman who has a straightforward labour and vaginal birth.

Risk to the woman

……………………………………………………………..

Minimal Extreme risk risk

Risk to the baby

……………………………………………………………..

Minimal Extreme risk risk

1. A woman over 42 weeks pregnant who declines induction of labour.

Risk to the woman

……………………………………………………………..

Minimal Extreme risk risk

Risk to the baby

……………………………………………………………..

Minimal Extreme risk risk

The following statements ask you to think about how other people think about risk in pregnancy and childbirth compared to you. Please circle the statements you most agree with.

1. In general, other doctors working in obstetrics think pregnancy and birth are…

| A lot less risky than I do. | Somewhat less risky than I do. | A little less risky than I do. | They agree with me about the degree of risk involved. | A little more risky than I do. | Somewhat more risky than I do. | A lot more risky than I do. |
| --- | --- | --- | --- | --- | --- | --- |

1. In general, midwives think pregnancy and birth are…

| A lot less risky than I do. | Somewhat less risky than I do. | A little less risky than I do. | They agree with me about the degree of risk involved. | A little more risky than I do. | Somewhat more risky than I do. | A lot more risky than I do. |
| --- | --- | --- | --- | --- | --- | --- |

1. In general, pregnant women think pregnancy and birth are…

| A lot less risky than I do. | Somewhat less risky than I do. | A little less risky than I do. | They agree with me about the degree of risk involved. | A little more risky than I do. | Somewhat more risky than I do. | A lot more risky than I do. |
| --- | --- | --- | --- | --- | --- | --- |

If you would like to receive an electronic copy of the results of the study, please tick the box. Please provide an email address………………………………………………………………..
