# Supplementary material for: Pregnancy related risk perception in pregnant women, midwives & doctors: a cross-sectional survey
Source: BMC Pregnancy Childbirth. 2019 Sep 27;19:335. doi: 10.1186/s12884-019-2467-4 (PMC6764151; doi:10.1186/s12884-019-2467-4)
Supplement: Supplementary file 2 — Differences between perceived risk ratings by pregnant women, midwives and doctors for individual scenarios. (DOCX 14 kb) [file 12884_2019_2467_MOESM2_ESM.docx]

**Supplementary File 2: Differences between perceived risk ratings by pregnant women, midwives and doctors for individual scenarios**

| Scenario | Risk to woman  Significance^1^ | Risk to baby  Significance^1^ |
| --- | --- | --- |
| A woman in labour being cared for by an experienced midwife | .024* | .001* |
| A women who gives birth on all fours | <.000* | .000* |
| A woman having a caesarean section because her labour did not progress | .013* | .000* |
| A pregnant woman with a supportive partner | .004* | .006* |
| A pregnant woman experiencing domestic violence | .004* | .000* |
| A woman who chooses to give birth with just her partner present at home | .101 | .724 |
| A woman who has a minor postpartum haemorrhage at home | <.000* | .000* |
| A pregnant 13 year old | .205 | .393 |
| A pregnant woman who does not attend any antenatal care | <.000* | .002* |
| A woman with a complicated labour being cared for a by a junior doctor | <.000* | .001* |
| A pregnant woman who is financially wealthy | .010* | .003* |
| A woman with a shoulder dystocia in hospital | <.000* | .016* |
| A woman bleeding heavily at 37 weeks of pregnancy | .042* | .014* |
| A pregnant woman with mild pre-eclampsia not receiving any antenatal care | .001* | .001* |
| A woman giving birth in a birthpool in hospital | .038* | .064 |
| A woman with a complicated pregnancy being cared for by a junior doctor | <.000* | .000* |
| A woman in labour being cared for by a newly qualified midwife | .023* | .022* |
| A woman who is very anxious during her pregnancy | .003* | .000* |
| A woman bleeding heavily at 24 weeks of pregnancy | <.000* | .440 |
| A pregnant 38 year old | <.000* | .000* |
| A woman who has a minor postpartum haemorrhage in hospital | <.000* | .000* |
| A woman with a shoulder dystocia at home | .025* | .051 |
| A woman who begins her pregnancy 3 stone (19kg) overweight | .086 | .072 |
| A woman in her first pregnancy giving birth in hospital. The pregnancy has been straightforward | .337 | .220 |
| A woman with a cord prolapse at home. | <.000* | .000* |
| A woman who plans to give birth vaginally to a breech baby at home | .002* | .160 |
| A woman whose labour is induced | <.000* | .000* |
| A woman who has an epidural in labour | <.000* | .028* |
| A woman in her second pregnancy giving birth in hospital. The pregnancy has been straightforward | .039* | .027* |
| A woman who gives birth lying on her back | <.000* | .000* |
| A woman who gives birth at 26 weeks of pregnancy | <.000* | .391 |
| A woman with a complicated labour being cared for by a consultant obstetrician | .012* | .068 |
| A woman in her first pregnancy giving birth at home. The pregnancy has been straightforward | <.000* | .000* |
| A woman who breastfeeds her baby | .002* | .012* |
| A woman who has a major postpartum haemorrhage at home | .032* | .000* |
| A woman who gives birth standing up | <.000* | .000* |
| A woman with a cord prolapse in hospital | <.000* | .000* |
| A pregnant woman who is financially poor | .008* | .001* |
| A woman who develops diabetes in pregnancy and follows the advice of the hospital healthcare team regarding diet and insulin | <.000* | .000* |
| A woman who gives birth at 34 weeks of pregnancy | .001* | .002* |
| A woman having a caesarean section because she wants to plan her baby’s birth date | <.000* | .000* |
| A pregnant woman receiving antenatal care from an experienced community midwife | .028* | .034* |
| A woman who plans to give birth vaginally to a breech baby in hospital | .005* | .331 |
| A pregnant woman who attends regular antenatal appointments | .008* | .038* |
| A pregnant woman with severe pre-eclampsia not receiving any antenatal care | .008* | .000* |
| A pregnant 45 year old | .003* | .000* |
| A woman who is depressed during her pregnancy | .038* | .195 |
| A woman who bottle-feeds her baby | .003* | .000* |
| A woman having a caesarean section because her obstetrician has recommended it | <.000* | .002* |
| A woman planning a vaginal birth of twins at home | .003* | .001* |
| A woman who begins her pregnancy 1stone (6.4kg) overweight | .142 | .194 |
| A woman who has a caesarean section because her baby is breech | <.000* | .000* |
| A pregnant woman with severe pre-eclampsia being treated in hospital | .358 | .556 |
| A woman who has a forceps delivery | .012* | .000* |
| A pregnant asylum seeker | <.000* | .000* |
| A pregnant woman who does not have many friends | <.000* | .000* |
| A woman in labour who does not speak English | .004* | .001* |
| A woman with a complicated pregnancy being cared for by a consultant obstetrician | .011* | .016* |
| A woman giving birth in a birthpool at home | <.000* | .000* |
| A woman with a retained placenta at home following a homebirth | .151 | .001* |
| A woman who experiences postnatal depression after giving birth | .191 | .117 |
| A pregnant woman without a partner | .080 | .151 |
| A woman planning a vaginal birth of twins in hospital | .003* | .001* |
| A woman who experiences the ‘baby blues’ after giving birth | .034* | .277 |
| A woman who has a Syntocinon drip in labour | <.000* | .000* |
| A woman who chooses to give birth alone at home | .576 | .734 |
| A pregnant woman who has close supportive friends | .077 | .197 |
| A pregnant woman with mild pre-eclampsia having regular appointments with her midwife and obstetrician | .006* | .012* |
| A woman planning a homebirth who gives birth alone because her labour is so fast and the midwife has not arrived | <.000* | .000* |
| A woman who has a major postpartum haemorrhage in hospital | .001* | .001* |
| A woman who develops diabetes in pregnancy and does not follow the advice of the hospital healthcare team regarding her diet | <.000* | .002* |
| A woman who experiences post-traumatic stress disorder after giving birth | <.000* | .035* |
| A woman having a caesarean section for twins | <.000* | .000* |
| A pregnant 17 year old | .242 | .278 |
| A woman who develops diabetes in pregnancy and does not follow the advice of the hospital healthcare team regarding taking insulin | .001* | .111 |
| A woman with a retained placenta following a hospital birth | <.000* | .000* |
| A woman in her second pregnancy giving birth at home. The pregnancy has been straightforward | <.000* | .000* |
| A woman who has a ventouse delivery | <.000* | .000* |
| A woman who has a straightforward labour and vaginal birth | .001* | .000* |
| A woman over 42 weeks pregnant who declines induction of labour | <.000* | .117 |

Kruskal-Wallis test *p<.05
